# Mental Health Outcomes in Perinatal Women During the Remission Phase of COVID-19 in China

**DOI:** 10.3389/fpsyt.2020.571876

**Published:** 2020-10-06

**Authors:** Xiaoqin Zeng, Wengao Li, Hengwen Sun, Xian Luo, Samradhvi Garg, Ting Liu, Jingying Zhang, Yongfu Zhang

**Affiliations:** ^1^Department of Gynecology, Guangzhou Women and Children’s Medical Centre, Guangzhou, China; ^2^Department of Psychiatry, Guangdong 999 Brain Hospital, Guangzhou, China; ^3^Department of Radiotherapy, Cancer Center, Guangdong Provincial People’s Hospital (Guangdong Academy of Medical Sciences), Guangzhou, China; ^4^Department of Psychiatry, Southern Medical University Nanfang Hospital, Guangzhou, China; ^5^School of Health in Social Science, University of Edinburgh, Edinburgh, United Kingdom; ^6^Department of Anesthesiology, Guangzhou Women and Children’s Medical Centre, Guangzhou, China

**Keywords:** China, COVID-19, mental health, perinatal, women

## Abstract

**Background:**

Since the middle of March, the COVID-19 outbreak has been well contained in China. The prevention and control measures for the outbreak have been downgraded to a normalized level. However, until now, the change in level of psychological health amongst perinatal women during the remission phase of the COVID-19 outbreak has not been investigated in China. The aim of this current study was to assess the symptoms of anxiety, depression, insomnia and quality of life (QOL) in perinatal women and to identify potential risk factors associated with these symptoms.

**Methods:**

This was a cross-sectional, hospital-based survey conducted between March 25^th^ till June 5^th^, 2020 in southern China. Convenient sampling method was adopted. Women’s anxiety, depression, insomnia symptoms and QOL was examined through standardized measurements. Multivariate logistic regression and Analysis of Covariance (ANCOVA) was conducted for the same.

**Results:**

A total of 625 perinatal women completed the study; of them, 195 women (31.2%, 95%CI=27.56%–34.84%) reported anxiety, 120 (19.2%, 95%CI=16.10%–22.30%) reported depression, and 87 (13.9%, 95%CI=11.20%–16.64%) experienced symptoms of insomnia. Previous adverse experiences during pregnancy was a significant risk factor for anxiety (OR=1.628, 95%CI=1.069–2.480, P=0.023), depression (OR=1.853, 95%CI=1.153–2.977, P=0.011), and insomnia (OR=2.160, 95%CI=1.290-3.616, P=0.003). Participants having infected friends/families/colleagues were more likely to report anxiety (OR=2.195, 95%CI=1.245–3.871, P=0.007) and depression (OR=2.666, 95%CI=1.482–4.794, P=0.001). Those women whose regular check-ups were severely interrupted by the COVID-19 were also more likely to experience symptoms of anxiety (OR=2.935, 95%CI=1.701–5.062, P<0.001) and insomnia (OR=2.195, 95%CI=1.098–4.390, P=0.026).

**Conclusion:**

The COVID-19 pandemic does affect the mental health and well being of perinatal women. Increased attention should be paid to women who have infected friends/families/colleagues and those with previous adverse experiences during pregnancy. Coping strategies that relieve psychological stress during the COVID-19 outbreak should be provided to prevent adverse outcomes for women and their infants.

## Introduction

Since the outbreak of the novel coronavirus disease (COVID-19) in late December in China, the authorities have undertaken effective measures to limit the rapid transmission of the disease (1). For example, most cities have set up emergency hospitals and quarantine facilities; public services and transportation have been strictly restricted and mandatory quarantine procedures have been adopted for individuals with suspected or confirmed diagnosis (2). However, in the meantime, these stringent control policies have interrupted the delivery of routine medical care for certain vulnerable populations, consisting of pregnant and postnatal women.

The ever increasing numbers of confirmed cases and deaths, inconvenient public transportation, barriers to access maintenance treatment, decreased healthcare provision, and elevated risk of infection when frequenting clinics/hospitals has exacerbated the psychological stress among certain vulnerable sub-populations, such as pregnant women, children, older adults, and chronic patients with mental illness (1, 3, 4). The consequences of mass quarantine and subsequent control measures to curb the spread of the viral epidemic has also highlighted huge challenges while delivering health care to this aforementioned population, however, little empirical evidence have been provided.

Pregnancy is characterized by significant hormonal and anatomical changes, which often leads to multiple kinds of psychological disturbances (5). Nevertheless, mental illness in pregnant and postpartum women has often been unrecognized and underestimated as it has parallels with certain somatic symptoms, such as fatigue, headache, loss of appetite, and energy (6). It has been consistently reported that advanced maternal age, intimate violence, lower economic level, and unwanted pregnancy were significantly associated with mental disturbances in pregnant women (7–10). In contrast, comfortable and secure living environment, family, and social support, as well as psychological resilience were meaningful buffers against psychological disturbances in pregnant and postpartum women (11).

As a significant life-threatening public health event, the COVID-19 pandemic deteriorates the mental health status of perinatal women, especially those with deficient support systems (e.g., limited contact with family and friends) (12). A recent study found that more than 1/3 of the perinatal women experienced depression during the outbreak (13), and pregnant women reported a higher rate of depression after the declaration of the COVID-19 pandemic (29.6% vs. 26.0%) (14). Besides increased depressive symptoms, perinatal women also reported elevated symptoms of anxiety, dissociation, post-traumatic stress disorder, as well as sleep disturbances during the COVID-19 pandemic (15).

A number of the pregnant and postpartum women also expressed fear of being infected with COVID-19, and it has been shown that the fear of COVID-19 was significantly related to depression, anxiety, suicidal ideation, and poor psychological quality of life (QOL) (12). Luckily, current studies suggest that there is no direct evidence for intrauterine infection caused by vertical transmission in women who develop COVID-19 pneumonia in their third trimester (16). Meanwhile, it seems that pregnancy and childbirth tend to not aggravate the course of symptoms or CT features of COVID-19 pneumonia (17). Existing evidence also indicated that timely termination of pregnancy would not increase the likelihood of premature birth and asphyxia of the newborn (18).

Since the middle of March, the COVID-19 outbreak has been well contained in China (19). The prevention and control measures for the outbreak have been downgraded to normalized measures. However, little is known about the mental health responses of perinatal women after the peak of the outbreak. Until now, the changes in level of psychological health among perinatal women during the remission phase of the COVID-19 outbreak have not been investigated. Therefore, the aim of the current study was to: (1) assess the prevalence of symptoms of anxiety, depression, insomnia in perinatal women after the peak of COVID-19 outbreak in China; (2) identify potential sociodemographic and clinical risk factors associated with these symptoms; and (3) examine the association between mental health status and QOL. Considering the adverse consequences of COVID-19 on both individual’s physical and psychological health, we hypothesized that: (1) the prevalence of disturbing mental health symptoms during the remission phase of the pandemic would be lower than before; (2) COVID-19 relevant factors, such as interrupted medical check-ups would be significantly associated with poorer mental health status; and (3) poor mental health status (such as depression, anxiety, and insomnia) would be significantly linked to impaired QOL.

## Methods

### Study Design

This is a cross-sectional, hospital-based survey conducted from March 25^th^ to June 5^th^, 2020 in southern China. Data were collected from Guangzhou Women and Children’s Medical Centre, the Provincial People’s Hospital, and Southern Medical University Nanfang Hospital. Convenient sampling method was adopted. To be eligible, participants were: (1) adults (aged 18 or above); (2) perinatal women (from the 28th week of pregnancy to one week after childbirth); (3) able to understand Mandarin and/or Cantonese; and (4) willing to provide written informed consent. Participants were excluded if they had pre-existing psychiatric disorders and/or disturbance of consciousness.

Eligibility checks were conducted by trained research staff at the time of enrolment of participants. For those who provided written informed consent, a face-to-face interview was conducted by a senior physician/nurse who was responsible for on-site recruitment and evaluation. Women were asked to complete a socio-demographic data collection form, and a set of standardized scales to assess their symptoms related to anxiety, depression, insomnia, and QOL. Ethical approval was obtained from Guangzhou Women and Children’s Medical Centre (ID: 2020-29801). Written informed consent was provided by all participants prior to their enrolment. Participants were allowed to terminate the study at any time they desired. The study was anonymous. Confidentiality of information was assured to all.

### Instruments

#### Sociodemographic and Clinical Information Sheet

A specially-designed sociodemographic and clinical information sheet was used to collect data, such as, participant’s age, education level, marital status, employment status, pregnancy, multiparty status, and previous medical history. Women were also asked to answer: (1) whether they have any friends, families, or colleagues who have been infected by the COVID-19? (Yes/No)? (2) whether the COVID-19 outbreak affected their regular medical check-ups in hospital (Not at all/moderately affected/severely affected); (3) Have they ever experienced any adverse pregnancy events previously, such as natural miscarriage (Yes/No)? (4) have they currently been diagnosed with any pregnancy-related comorbidities, such as pregnancy hypertension, and gestational diabetes (Yes/No)? and (5) whether they are suffering from any other physical comorbidities, such as musculoskeletal or neurological disease (Yes/No)?

#### General Anxiety Disorder Questionnaire (GAD)

The 7-item GAD is a self-report scale used to evaluate an individual’s anxiety symptoms. Response options available are rated from 0 to 3, and a sum score of 5 or more indicates anxiety symptoms (20). The GAD has been translated and well-validated in the Chinese language. It has excellent psychometric properties (internal consistency=0.91) (21).

#### Edinburgh Postnatal Depression Scale (EPDS)

The 10-item EPDS is a self-reported measure to assess the severity of depressive symptoms in the last seven days of pregnant and postpartum women (22). Its total score ranges from 0 to 30, with a higher score indicating more severe depressive symptoms. A sum score of 13 or above indicates severe depressive symptoms. The Chinese version of EPDS showed satisfactory psychometric properties (23).

#### Insomnia Symptoms

Three standardized questions based on the Diagnostic and Statistical Manual of Mental Disorders (DSM-IV) were used to measure an individual’s insomnia symptoms in the last month (24), namely: (1) difficulties initiating sleep (DIS) - Do you take more than 2 hours to fall asleep nearly every night? (2) difficulties in maintaining sleep (DMS) - Do you wake up during your sleep and take 1 hour or more to get back to sleep nearly every night? and (3) Early morning awakening (EMA) - Do you wake up nearly every morning at least 2 hours earlier than you wanted to? All three questions were Yes/No questions (No=0, and Yes=1). A total score of one or more were considered as having insomnia symptoms.

#### World Health Organization Quality of Life Questionnaire (WHOQOL-BREF)

The 26-item WHOQOL-BREF questionnaire was used to assess a woman’s quality of life, covering four aspects, namely: physical, psychological, environmental, and social domains (25). A higher total score on this scale indicates a higher QOL (26). The Chinese version of the scale has good psychometric properties (27).

### Sample Size Estimation

Sample size of participants was estimated by a standardized formula: N = Z_α_^2^ P (1 − P)/*d*^2^ (4). As recommended, we set α (significance level) as 0.05, Z_α_ (statistic of significance test) as 1.96, and the estimated acceptable margin of effort for proportion *d* was 0.1 (28). According to previous systematic review and meta-analysis, the overall prevalence of perinatal depression was around 15% (29–31). Thus, the P (Prevalence) was set as 0.15 in the current study. To improve statistical power (4), the targeted sample size was amplified by 20%. Finally, the minimum sample size for this study was set at 588.

### Statistical Analyses

All data were input in the Epidata 3.0 (Epidata Association, Odense, Denmark), and data analyses were performed by SPSS 22.0 (IBM Corp). Normal distribution assumption was tested first by Kolmogorov-Smirnov test. Sociodemographic and clinical variables between the two groups was investigated using independent sample t-test (for normally distributed continuous data), Chi-square test (for categorical variables) or Mann-Whitney U test (for non-normally distributed continuous data). Bonferroni adjustment method was used for correction for multiple comparisons.

To further determine potential risk factors indicating symptoms of anxiety, depression and insomnia, all variables that were significant in the univariate analyses were further examined by multivariate logistic regression, and the associations between risk factors and outcomes were presented as odds ratios (ORs) and 95% Confidence Intervals (CIs). Symptoms of anxiety, depression, and insomnia were the dependent variable, whilst variables which were significant in univariate group differences were entered as independent variables. To further explore the association between mental health status (anxiety, depression and insomnia symptoms) and QOL, analysis of covariance (ANCOVA) was conducted. Anxiety, depression and insomnia (all Yes/No categorical variables) were entered as independent variable, while QOL was entered as dependent variable. Variables with significant difference in univariate analyses were controlled as covariates. Significance was set at 0.05, with two-tailed tests.

## Results

### Sociodemographic and Clinical Characteristics

In total, 868 eligible women were invited to participate in the study, and eventually 625 (72.0%) signed the informed consent form and completed the survey. Of these, 516 (82.6%) women were in their third trimester, while the remaining 109 (17.4%) were in postnatal period. The mean age of the participants was 29.19 years (SD=4.20), ranged from 18 to 47. Most of the women lived in an urban area (n=533, 85.3%), had high educational background (undergraduate and above, n=409, 65.6%), and were currently employed (n=364, 58.2%). More than 80% of them reported no previous adverse pregnancy experiences (n=504, 80.6%), no pregnancy-related illness (n=578, 92.5%), and no other physical comorbidities (n=537, 85.9%).

The majority of the participants had no friends, and/or families who were infected by the virus (n=567, 90.7%). In all, 141 (22.6%) of the women reported that their medical check-ups were not interrupted at all by the COVID-19 outbreak, 330 (52.8%) of them experienced moderate interruption, while 154 (24.6%) reported that their routine obstetric check-ups were severely affected by the outbreak. Anxiety symptoms was reported by 195 (31.2%) women (95%CI=27.56%–34.84%). Depressive symptom was reported by 120 (19.2%) women (95%CI=16.10%–22.30%), and 87 (13.9%) of them suffered from sleep disturbances (95%CI=11.20%–16.64%). Participants characteristics are summarized in **Table 1**.

**Table 1:** Characteristics of the Chinese perinatal women (N=625).

Variable	Total (N=625)	Anxiety				Depression				Insomnia			
	N (%)	No	Yes	X^2^/Z	*P*	No	Yes	X^2^/Z	*P*	No	Yes	X^2^/Z	*P*
**Pregnancy**													
Third Trimester	516 (82.6)	355 (56.8)	161 (25.8)			407 (65.1)	109 (17.4)			439 (70.2)	77 (12.3)		
Postnatal	109 (17.4)	75 (12.0)	34 (5.4)	0.001	0.999	98 (15.7)	11 (1.8)	7.060	**0.008**	99 (15.8)	10 (1.6)	2.481	0.115
**Urban Residents**													
No	92 (14.7)	64 (10.2)	28 (4.5)			77 (12.3)	15 (2.4)			79 (12.6)	13 (2.1)		
Yes	533 (85.3)	366 (58.6)	167 (26.7)	0.029	0.864	428 (68.5)	105 (16.8)	0.583	0.445	459 (73.4)	74 (11.8)	0.004	0.950
**Having Infected Friends/families, etc**													
No	567 (90.7)	403 (64.5)	164 (26.2)			469 (75.0)	98 (15.7)			493 (78.9)	74 (11.8)		
Yes	58 (9.3)	27 (4.3)	31 (5.0)	14.743	**<0.001**	36 (5.8)	22 (3.5)	14.459	**<0.001**	45 (7.2)	13 (2.1)	3.849	**0.049**
**COVID-19 Affected Regular Check-ups**													
Not at all	141 (22.6)	115 (18.4)	26 (4.2)			120 (19.2)	21 (3.4)			127 (20.3)	14 (2.2)		
Moderately	330 (52.8)	229 (36.6)	101 (16.2)			261 (41.8)	69 (11.0)			291 (46.6)	39 (6.2)		
Severely	154 (24.6)	86 (13.8)	68 (10.9)	22.792	**<0.001**	124 (19.8)	30 (4.8)	2.315	0.314	120 (19.2)	34 (5.4)	11.644	**0.003**
**Education level**													
High school and below	216 (34.6)	146 (23.4)	70 (11.2)			174 (27.8)	42 (6.7)			181 (29.0)	35 (5.6)		
Undergraduate	346 (55.4)	241 (38.6)	105 (16.8)			280 (44.8)	66 (10.6)			304 (48.6)	42 (6.7)		
Postgraduate and above	63 (10.1)	43 (6.9)	20 (3.2)	0.273	0.873	51 (8.2)	12 (1.9)	0.013	0.994	53 (8.5)	10 (1.6)	2.057	0.358
**Employment**													
No	261 (41.8)	179 (28.6)	82 (13.1)			212 (33.9)	49 (7.8)			223 (35.7)	38 (6.1)		
Yes	364 (58.2)	251 (40.2)	113 (18.1)	0.010	0.921	293 (46.9)	71 (11.4)	0.052	0.819	315 (50.4)	49 (7.8)	0.153	0.696
**Individual Monthly Income**													
less than 3000 RMB	142 (22.7)	108 (17.3)	34 (5.4)			112 (17.9)	30 (4.8)			116 (18.6)	26 (4.2)		
3,001-4,999 RMB	210 (33.6)	139 (22.2)	71 (11.4)			167 (26.7)	43 (6.9)			185 (29.6)	25 (4.0)		
5,000-10,000 RMB	193 (30.9)	129 (20.6)	64 (10.2)			163 (26.1)	30 (4.8)			172 (27.5)	21 (3.4)		
more than 10,001 RMB	80 (12.8)	54 (8.6)	26 (4.2)	4.558	0.207	63 (10.1)	17 (2.7)	2.440	0.486	65 (10.4)	15 (2.4)	6.041	0.110
**First Delivery**													
No	250 (40.0)	163 (26.1)	87 (13.9)			207 (33.1)	43 (6.9)			213 (34.1)	37 (5.9)		
Yes	375 (60.0)	267 (42.7)	108 (17.3)	2.516	0.113	298 (47.7)	77 (12.3)	1.074	0.300	325 (52.0)	50 (8.0)	0.269	0.604
**Past Adverse Pregnancy Experiences**													
None	504 (80.6)	359 (57.4)	145 (23.2)			418 (66.9)	86 (13.8)			445 (71.2)	59 (9.4)		
Yes	121 (19.4)	71 (11.4)	50 (8.0)	7.162	**0.007**	87 (13.9)	34 (5.4)	7.660	**0.006**	93 (14.9)	28 (4.5)	10.646	**0.001**
**Pregnancy-related Comorbidities**													
None	578 (92.5)	399 (63.8)	179 (28.6)			469 (75.0)	109 (17.4)			501 (80.2)	77 (12.3)		
Yes	47 (7.5)	31 (5.0)	16 (2.6)	0.191	0.662	36 (5.8)	11 (1.8)	0.579	0.447	37 (5.9)	10 (1.6)	2.295	0.130
**Other Physical Comorbidities**													
None	537 (85.9)	374 (59.8)	163 (26.1)			444 (71.0)	93 (14.9)			469 (75.0)	68 (10.9)		
Yes	88 (14.1)	56 (9.0)	32 (5.1)	1.272	0.259	61 (9.8)	27 (4.3)	8.704	**0.003**	69 (11.0)	19 (3.0)	5.030	**0.025**
**Pregnancy Secondhand Smoking**													
None	472 (75.5)	334 (53.4)	138 (22.1)			384 (61.4)	88 (14.1)			408 (65.3)	64 (10.2)		
Yes	153 (24.5)	96 (15.4)	57 (9.1)	3.460	0.063	121 (19.4)	32 (5.1)	0.384	0.535	130 (20.8)	23 (3.7)	0.209	0.647
	**Total (N=625)**	**Anxiety**				**Depression**				**Insomnia**			
	**Mean (SD)**	**No**	**Yes**	**T**	***P***	**No**	**Yes**	**T**	***P***	**No**	**Yes**	**T**	***P***
**Age (years)**	29.2 (4.2)	29.2 (4.2)	29.3 (4.2)	-0.231	0.817	29.2 (4.2)	29.1 (4.3)	0.137	0.891	29.2 (4.2)	29.2 (4.1)	0.081	0.936
**BMI**	25.4 (3.8)	25.3 (3.9)	25.5 (3.6)	-0.402	0.688	25.4 (3.8)	25.3 (3.9)	0.238	0.812	25.3 (3.8)	25.6 (3.7)	-0.498	0.618
**Physical QOL**	15.1 (2.1)	15.2 (2.0)	15.0 (2.1)	0.784	0.433	15.5 (1.9)	13.7 (2.2)	8.679	**<0.001**	15.3 (2.0)	14.5 (2.1)	3.336	**0.001**
**Psychological QOL**	15.3 (2.5)	15.4 (2.4)	15.1 (2.7)	1.101	0.272	15.8 (2.3)	13.3 (2.4)	10.903	**<0.001**	15.4 (2.5)	14.7 (2.4)	2.379	**0.018**
**Social QOL**	15.5 (2.4)	15.5 (2.4)	15.4 (2.5)	0.429	0.668	15.9 (2.2)	13.7 (2.4)	8.983	**<0.001**	15.6 (2.5)	14.8 (2.2)	2.921	**0.004**
**Environmental QOL**	15.1 (2.5)	15.1 (2.4)	14.9 (2.7)	0.825	0.410	15.5 (2.3)	13.0 (2.4)	10.611	**<0.001**	15.1 (2.5)	14.5 (2.6)	2.206	**0.028**

BMI, Body mass index; EPQS, Edinburgh Postnatal Depression Scale; QOL, Quality of life; In bold: P<0.05.

### Univariate Analysis

Univariate analysis showed that women who had infected friends/families/colleagues, and previous adverse pregnancy experiences were significantly associated with anxiety (P<0.001, and P=0.007), depressive (P<0.001, and P=0.006), and insomnia symptoms (P=0.049, and P=0.001). Routine check-ups being interrupted by COVID-19 was also significantly associated with anxiety (P<0.001) and insomnia (P=0.003), while other physical comorbidities was associated with depression (P=0.003) and insomnia symptoms (P=0.025). Additionally, women in their third trimester (P=0.008) were more likely to report depression than postnatal women. Independent sample t-test showed that women with depressive and insomnia symptoms reported lower QOL in all four domains compared to those without (P all<0.05). However, the differences were not significant between the anxiety group and no-anxiety group (see **Table 1**).

### Multivariate Analysis

Multivariate analysis confirmed that previous adverse pregnant experience was a significant risk factor for anxiety (OR=1.628, P=0.023), depression (OR=1.853, P=0.011), and insomnia (OR=2.160, P=0.003). Women who have positively infected friends/families/colleagues were more likely to report symptoms of anxiety (OR=2.195, P=0.007) and depression (OR=2.666, P=0.001), and those whose regular check-ups were severely interrupted by the COVID-19 were more likely to experience symptoms of anxiety (OR=2.935, P<0.001) and insomnia (OR=2.195, P=0.026). Additionally, compared to women in their third trimester, postnatal women were less likely to suffer from depressive symptoms (OR=0.479, P=0.032). Women who reported other physical comorbidities had an increased likelihood of reporting depressive symptoms (OR=1.874, P=0.018) (see **Table 2**).

**Table 2:** Correlates of mental health outcomes by multivariate logistic regression analysis.

Variable	Multivariate logistic regression					*P*
Anxiety	Exp(B)	B	S.E.	95%CI lower	95%CI upper	
Having infected friends/families/colleagues	2.195	0.786	0.289	1.245	3.871	**0.007**
Regular check-up being affected by COVID-19						
Not at all	Ref	–	–	–	–	**-**
Moderately affected	1.799	0.587	0.251	1.101	2.941	**0.019**
Severely affected	2.935	1.077	0.278	1.701	5.062	**<0.001**
Past adverse pregnancy experiences	1.628	0.487	0.215	1.069	2.480	**0.023**
**Depression**						
Pregnancy						
Third Trimester	Ref	–	–	–	–	–
Postnatal	0.479	-0.735	0.343	0.245	0.939	**0.032**
Having infected friends/families/colleagues	2.666	0.980	0.299	1.482	4.794	**0.001**
Past adverse pregnancy experiences	1.853	0.617	0.242	1.153	2.977	**0.011**
Other physical comorbidities	1.874	0.628	0.266	1.112	3.158	**0.018**
**Insomnia**						
Having infected friends/families/colleagues	1.459	0.378	0.357	0.725	2.936	0.289
Regular check-up being affected by COVID-19						
Not at all	Ref	–	–	–	–	–
Moderately affected	1.098	0.093	0.335	0.570	2.115	0.780
Severely affected	2.195	0.786	0.353	1.098	4.390	**0.026**
Past adverse pregnancy experiences	2.160	0.770	0.263	1.290	3.616	**0.003**
Other physical comorbidities	1.708	0.536	0.298	0.952	3.065	0.072

In bold: P<0.05; CI, Confident Interval; COVID-19, Novel Coronavirus Disease.

ANCOVA analysis revealed that women with depressive symptoms reported lower physical, psychological, social and environmental QOL compared to those who without (P all <0.001) after controlling for covariates. Women with insomnia symptoms reported lower physical (P=0.004), psychological (P=0.041), and social (P=0.013) QOL compared to those without. However, there was no difference in environmental QOL between insomnia group and no-insomnia group after controlling for covariates (see **Table 3**).

**Table 3:** ANCOVA analysis.

ANCOVA	Depression					Insomnia				
	No	Yes	F	df	*P*	No	Yes	F	df	*P*
Physical QOL	15.47 (1.90)	13.74 (2.18)	75.74	1	**<0.001**^a^	15.25 (2.05)	14.46 (2.13)	8.336	1	**0.004**^b^
Psychological QOL	15.78 (2.25)	13.26 (2.36)	112.80	1	**<0.001**^a^	15.39 (2.48)	14.71 (2.39)	4.203	1	**0.041**^b^
Social QOL	15.89 (2.23)	13.71 (2.43)	80.67	1	**<0.001**^a^	15.59 (2.45)	14.77 (2.16)	6.240	1	**0.013**^b^
Environmental QOL	15.53 (2.28)	13.04 (2.42)	108.61	1	**<0.001^a^**	15.14 (2.49)	14.50 (2.57)	3.226	1	0.073^b^

^a,^using pregnancy, having infected friends/families/colleagues, adverse pregnant experience, and other physical comorbidities as covariates; ^b,^using having infected friends/families/colleagues, regular check-ups being affected by COVID-19, adverse pregnant experience, and other physical comorbidities as covariates; QOL, Quality of Life; In bold: P<0.05; COVID-19, Novel Coronavirus Disease.

## Discussion

When facing a major life-threatening public health event, pregnant and postpartum women tend to experience more psychological disturbances due to additional concerns about their unborn fetus or babies (13). To the best of our knowledge, this was the first investigation that examined the mental health outcomes amongst Chinese perinatal women during the remission phase of the COVID-19 outbreak. In the current study, the prevalence of anxiety, depressive, and insomnia symptoms was 31.2%, 19.2%, and 13.9%, respectively, amongst perinatal women.

The prevalence of depression found in the current study was lower than the results found in previous studies using the same EPDS measurement amongst pregnant women during the peak of COVID-19 outbreak (34.2% in China, and 35.4% in Turkey) (13, 14). The prevalence of anxiety and insomnia symptoms found in this study was also lower than most of the findings reported within the general population during the peak of the COVID-19 epidemic (32, 33). Since mid-March, the Chinese government has lifted the lockdown policy in many cities, specific healthcare guidelines for perinatal women and children have been released, and newly confirmed domestic cases on the Chinese mainland have dropped considerably. China has earned initial victory in this critical battle and the public are more confident and calm in their fight against the epidemic. Additionally, the increasing healthcare budgets, easier access to obstetric services, and the improving clinical service quality have also contributed in relieving mental stress and alleviating COVID-19 related psychological distress in perinatal women.

After the announcement of the human-to-human transmission, and the declaration of lockdown policy on 23rd January, 2020 in Wuhan, China, there was a significant rise in the prevalence of anxiety, and depressive symptoms amongst pregnant women (1, 14). To reduce the adverse consequences of psychological disturbances reported within this population, experts suggested that screening should be implemented with adequate systems to ensure accurate diagnosis, effective treatment, and appropriate follow-up (34). In late February, the national health authorities and Chinese academic societies published specific expert consensus and intervention guidelines, appropriately labeled, “The Manual of Novel Coronavirus Pneumonia Prevention and Control for Children and Pregnant Women” (35). This manual was a helpful guide designed to alleviate the psychological stress of this aforementioned population and improve the quality of mental health services.

Previous studies have exhibited that strict management policies during the COVID-19 could worsen the pre-existing mental stress and increase the likelihood of experiencing negative emotions (36), such as fear, agitation, loneliness, interpersonal sensitivity, and some of these individuals may even develop impulsivity, harmful alcohol use, suicidal thoughts and PTSD symptoms (37). However, since mid-March, the prevention and control measures in most of the provinces in China have been downgraded to a normalized level. As of 5^th^ June, 2020, there are only 66 patients with COVID-19, and 2 suspicious cases in mainland China (38). With the ever decreasing number of confirmed cases and simultaneous deaths, easier access to obstetric healthcare services and the improved healthcare service quality is possible. It is therefore reasonable to speculate that the prevalence of mental disturbances among perinatal women in China would further descend in the future based on this trend.

In accordance with previous findings, we found that participants who have infected friends/families/colleagues were more likely to report anxiety and depressive symptoms. A study showed that the prevalence rate of depressive symptoms was positively associated with the number of newly-confirmed COVID-19 cases, suspected infections, and death cases per day in China (14). For pregnant women, it is possible that they were more worried about their increased risk of getting infected due to their naturally suppressed immune system during pregnancy. Consequently, their concerns about vertical transmission to their fetus may also contribute to their elevated psychological disturbances. However, it is worth noting that, in the current study, only less than 10% of the women had known someone who is infected with COVID-19. Longitudinal studies with a larger sample size are needed to better explore the association between these two variables.

In addition, during the COVID-19 epidemic, many hospitals in China were designated as temporary medical facilities for confirmed patients or suspected cases (39), which restricted the provision of obstetric services. However, routine office visits with physical examinations and routine lab tests are extremely important for pregnant women in screening for various complications during/after pregnancy. Due to limited maternity and gynecological services provision during the COVID-19 outbreak, pregnant women may therefore question the capability of local public health facilities to provide high-standard prenatal care and childbirth services. This could also be a potential reason explaining why those with interrupted routine check-ups were at higher risk of experiencing psychological disturbances. To better improve maternity services, women need to be ensured that they would have access to appropriate levels of care throughout the day and night. Effective, swift and safe transfers to centers of obstetric care should be guaranteed for the health of mother and infant.

In accordance with previous study’s findings, our study found that past adverse pregnancy experience was significantly associated with maternal mental health outcomes. A recent meta-analysis confirmed a significant association between perinatal loss, and increased anxiety and depression levels in subsequent pregnancies (40). A large-population-based longitudinal study reported that a prior miscarriage was associated with anxiety as well as depressive symptoms at the 14-year follow-up, and having had a neonatal, infant death was significantly associated with symptoms of depression (41). Different adverse pregnancy events are believed to increase the likelihood for a wide range of maternal mental health problems. These stressful experiences might trigger adverse psychological reaction and compromise women’s psychological adjustment.

In the field of QOL among perinatal women, the focus has expanded from simply “preventing, detecting and managing complications” to “supporting psychological adaptation” (42). Mental disturbances during pregnancy and postnatal period increase risk for both the mother and the infant, and greatly influence their QOL (43). Women who reported symptoms of anxiety, depression, and poor sleep quality tend to have higher chances of preterm birth, longer labor, more somatic discomforts, and poorer mother-infant attachment, which could result in decreased QOL (44–47). Studies have also indicated that worries, depression, tiredness, and perineal pain significantly compromise perinatal women’s QOL (48). Therefore, screening for perinatal psychological disturbances has been internationally recommended, and should be considered a priority during a public health crisis.

To summarize, several limitations should be acknowledged. Firstly, only perinatal women from southern China were invited to participate in the study. Most of these women were in their third trimester, and lived in an urban area, which might cause selection bias. Secondly, this was a cross-sectional study, the causal association between tested variables and mental disturbances could not be determined. Longitudinal studies are warranted to study the trajectory of mood symptoms amongst perinatal women during the COVID-19 epidemic. Thirdly, anxiety, depression and insomnia symptoms were assessed by self-report scales, rather than using standardized diagnostic instruments, such as the Structured Clinical Interview for DSM-IV (SCID). Further studies with more representative samples using validated objective instruments are needed to address this shortcoming. Finally, in the current study, we only included perinatal women from their 28^th^ week of pregnancy to one week after delivery. The timeframe is rather restrictive and may not be representative of the entire perinatal period.

In conclusion, the COVID-19 pandemic affects mental health outcomes amongst perinatal women. Our study indicated that increased attention should be paid to patients who have infected friends and/or families and those with previous adverse pregnancy experiences. Copying strategies targeting relieving psychological stress during the COVID-19 outbreak should be provided to prevent adverse outcomes for women and their infants. To better improve maternity services, routine prenatal care and childbirth services should be ensured. Effective, swift and safe transfers to centers of obstetric care should be guaranteed.

## Data Availability Statement

The raw data supporting the conclusions of this article will be made available by the authors, without undue reservation, to any qualified researcher.

## Ethics Statement

The studies involving human participants were reviewed and approved by Guangzhou Women and Children’s Medical Centre (ID:2020-29801). The patients/participants provided their written informed consent to participate in this study.

## Author Contributions

XZ and YZ were responsible for study design. XZ, WL, HS, TL, and JZ were responsible for data collection, analysis and interpretation. XZ, WL, and HS drafted the manuscript, YZ, SG, and XL revised the manuscript. All authors contributed to the article and approved the submitted version.

## Funding

This study is funded by the Guangzhou Science and Technology Project (201804010132), Traditional Chinese Medicine Bureau of Guangdong Province (20192076), and the President Foundation of Nanfang Hospital (2017L001).

## Conflict of Interest

The authors declare that the research was conducted in the absence of any commercial or financial relationships that could be construed as a potential conflict of interest.
